# Efficacy and safety of acupuncture for dizziness and vertigo in emergency department: a pilot cohort study

**DOI:** 10.1186/s12906-015-0704-6

**Published:** 2015-06-09

**Authors:** Chih-Wen Chiu, Tsung-Chieh Lee, Po-Chi Hsu, Chia-Yun Chen, Shun-Chang Chang, John. Y. Chiang, Lun-Chien Lo

**Affiliations:** Department of Emergency Medicine, Changhua Christian hospital, Changhua, Taiwan; Department of Chinese medicine, Changhua Christian hospital, Changhua, Taiwan; Graduate Institute of Statistical and informational Science, National Changhua University of Education, Changhua, Taiwan; Department of Computer Science and Engineering, National Sun Yat-sen University, Kaohsiung, Taiwan; Department of Healthcare Administration and Medical Informatics, Kaohsiung Medical University, Kaohsiung, Taiwan

**Keywords:** Dizziness and vertigo, Emergency department (ED), Clinical trial, Acupuncture, Traditional Chinese medicine (TCM)

## Abstract

**Background:**

Dizziness and vertigo account for roughly 4% of chief symptoms in the emergency department (ED). Pharmacological therapy is often applied for these symptoms, such as vestibular suppressants, anti-emetics and benzodiazepines. However, every medication is accompanied with unavoidable side-effects. There are several research articles providing evidence of acupuncture treating dizziness and vertigo but few studies of acupuncture as an emergent intervention in ED. We performed a pilot cohort study to evaluate the efficacy and safety of acupuncture in treating patients with dizziness and vertigo in ED.

**Methods:**

A total of 60 participants, recruited in ED, were divided into acupuncture and control group. Life-threatening conditions or central nervous system disorders were excluded to ensure participants’ safety. The clinical effect of treating dizziness and vertigo was evaluated by performing statistical analyses on data collected from questionnaires of Dizziness Handicap Inventory (DHI), Visual Analog Scale (VAS) of dizziness and vertigo, and heart rate variability (HRV).

**Results:**

The variation of VAS demonstrated a significant decrease (*p*-value: 0.001 and *p*-value: 0.037) between two groups after two different durations: 30 mins and 7 days. The variation of DHI showed no significant difference after 7 days. HRV revealed a significant increase in high frequency (HF) in the acupuncture group. No adverse event was reported in this study.

**Conclusion:**

Acupuncture demonstrates a significant immediate effect in reducing discomforts and VAS of both dizziness and vertigo. This study provides clinical evidence on the efficacy and safety of acupuncture to treat dizziness and vertigo in the emergency department.

**Trial registration:**

ClinicalTrials.gov ID: NCT02358239. Registered 5 February 2015

## Background

Dizziness and vertigo are one of the most challenging problems in the emergency department (ED), and roughly 4% of chief symptoms were reported [1, 2]. Dizziness and vertigo are commonly encountered diseases, which may be caused by many problems. Dizziness and vertigo can be triggered by peripheral vestibular disorders, central nervous system disorders, or combined lesions, as well as other conditions [3]. Some of these problems are not serious while others can be life-threatening, thus the first goal of clinicians hinges on identifying patients who need inpatient management or emergent intervention [4]. As a consequence, patients with dizziness undergo more diagnostic tests and have greater lengths of stay (LOS) than those without dizziness [5].

If there was no critical problem but the patient still suffered dizziness and vertigo, a physician would prescribe the medicine for symptoms relief and supportive care in ED. The treatment for dizziness and vertigo consists of only pharmacological therapy, such as vestibular suppressants, anti-emetics, and benzodiazepines in conventional medicine [6]. However, these medications are often accompanied with unavoidable side-effects and complementary therapies or medicines are always expected. Acupuncture has been used for relief of acute illness, such as pain, dizziness and vertigo in traditional Chinese medicine (TCM) over a thousand years. [7, 8] However, there has been relatively few research studies revealed acupuncture stimulation may induce an immediate effect to treat dizziness and vertigo [8, 9].

To the best of our knowledge, no study surveyed covered both conventional medicine and acupuncture to treat dizziness and vertigo in the emergency department (ED). The aim of our study focused on evaluating the efficacy and safety of acupuncture in patients with dizziness and vertigo in the emergency department (ED).

## Methods

### Ethics approval

A clinical pilot cohort study was conducted. The protocol identification number at http://www.clinicaltrials.gov was NCT02358239. All candidates received a standardized interview process. Participants were introduced of the purpose, procedures, potential risks and benefits of the study first, and signed the informed consent form. The trial was conducted from February to December, 2013. The protocol of the trial was approved by the Institutional Review Board of the Changhua Christian hospital, Changhua, Taiwan (IRB ref: 120403).

### Study subjects

Patients were recruited in the emergency department (ED) at Changhua Christian Hospital, Taiwan. All candidates went through a standardized interview process. The purpose, procedures, potential risks and benefits of the study were explained thoroughly to the participants. Participants were able to choose the experiment group or the control group to enroll study according to their willingness, and were able to withdraw from the study at any time without consequence.

### Inclusion criteria

Participants meeting the following criteria would be included:

1. Age 20 to 90 years, either gender.
2. Visit and stay in emergency department.
3. Consult otolaryngologist and neurologist to rule in dizziness and giddiness, auditory vertigo, vertebrobasilar artery syndrome, and peripheral vestibular disorders—Ménière’s disease, benign paroxymal peripheral vertigo, and vestibular neuritis.

### Exclusion criteria

Participants meeting one or more of the following criteria would be excluded:

1. Serious comorbid conditions, e.g., life-threatening condition or central nervous system disorder, including infarction, hemorrhage, and other vascular or structural disorders.
2. Patients who cannot communicate reliably with the investigator or who are not likely to obey the instructions of the trial.

### Sample size

Because there were few relevant previous studies available, we estimated an approximate sample size on the basis of the number of patients with dizziness visiting the emergency department (ED) of Changhua Christian hospital. We planned to enroll a total of 60 participants with 30 in each group to conduct this pilot study.

### Baseline assessment

#### Short form-36 quality-of-life questionnaire

The Short Form-36 Quality-of-Life Questionnaire (SF-36) is a widely used tool for evaluating a person’s health perception in daily life. SF-36 consists of 36 questions, grouped into eight health categories as follows: physical functioning (PF; 10 items); role limitation due to physical problems (RP; 4 items); bodily pain (BP; 2 items); vitality (VT; 4 items); general health perceptions (GH; 5 items); social functioning (SF; 2 items); role limitation due to emotional problems (RE; 4 items); and mental health (MH; 5 items). The standard SF-36 form asks questions with reference to the past 4 weeks [10, 11].

### Interventions

**Fig. 1:**
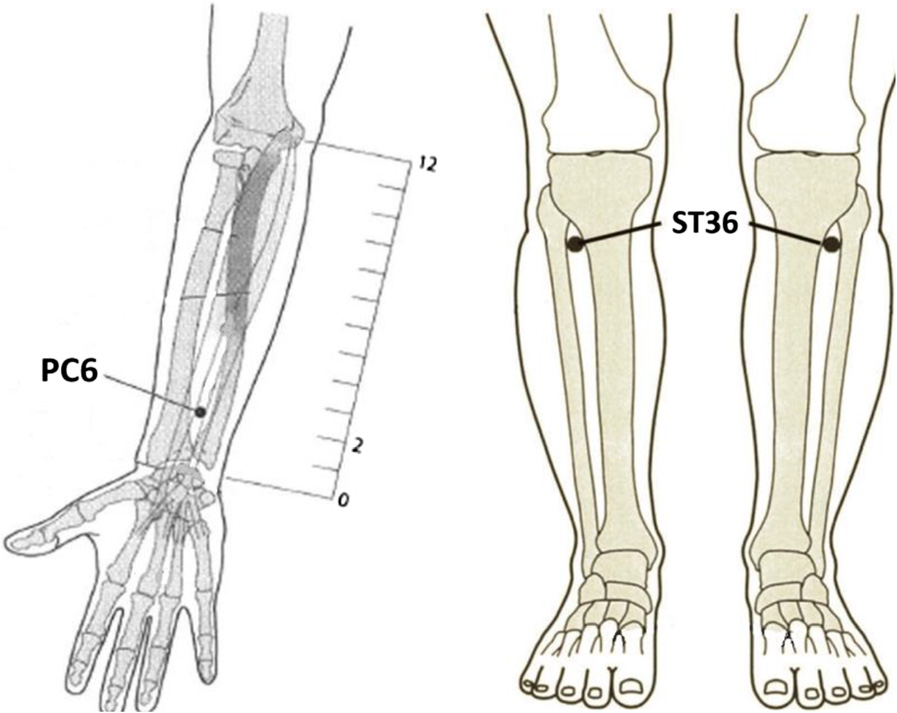
PC6 and ST36 acupoint locations

Patients meeting the inclusion criteria and none of the exclusion criteria were selected to control and experiment groups. Experiment group received acupuncture at Zusanli (ST36) and Neiguan (PC6) acupuncture points, *ref.* Fig. 1 [12]. Single-use, sterile, 40 mm × 0.25 mm, silver-handled needles (An-Chi disposable acupuncture needle, made in Taiwan) with guide tubes were used. Needles were inserted at correct acupoints and manually stimulated until the ‘De Qi’ sensation was elicited according to TCM protocol [13]. The needles stayed in place for 30 min. The control group received sham acupuncture by pasting seed-patches at non-acupoints. The non-acuponits were displaced by 1 cm from correct acupoints of experimental group. The therapist did not perform any massage or press stimulation to prevent acupressure effect.

### Primary outcome measures

#### Dizziness handicap inventory

The Dizziness Handicap Inventory (DHI) is a validated, self-reported questionnaire, which is widely used to evaluate the functional, emotional, and physical impact of dizziness on patients’ daily life. It consists of 25 questions regarding daily problems associated with dizziness, and each question is given in 3-item response scale, yes/sometimes/no, with the corresponding scored as 4/2/0 [14].

#### Visual Analog Scale (VAS) of dizziness and vertigo

The Visual Analog Scale (VAS), with proved usefulness and clinical validity for the evaluation of pain, is used as a measure of dizziness symptoms [15]. Advantages of VAS for the assessment of dizziness and vertigo include user-friendliness, rapidity, and adaptability to different cultures and languages [16].

#### Heart rate variability

Autonomic nervous dysfunction plays a role as a predisposing factor, a trigger or as a consequence of vertigo [17]. Recording the heart rate variability (HRV) is an easy, non-invasive method to investigate autonomic balance [18, 19]. In this work, a new ANSWatch wrist monitor (Taiwan Scientific Corporation, Taipei, Taiwan; Taiwan Department of Health medical device product registration number 001525), equipped with multiple piezo-electrical sensors enclosed in the cuff to directly measure the blood pressure waveforms in the radial artery, is used. Each ANSWatch test takes about 7 min and the parameters measured including heart rate variability (HRV), high frequency (HF), low frequency (LF), very low frequency (VLF), sympatho-parasympathetic balance index (LF/HF), total power, root mean square of successive differences (RMSSD), and the proportion of NN50 divided by total number of NNs (PNN50).

### Secondary outcome measures

#### Safety and length of stay

Participants should report any adverse events experienced, including discomfort or bruising at the sites of needle insertion, nausea, or feeling faint after treatment. Information regarding patient demographics, associated past history, trauma history, complications, and length of stay in ED were collected.

#### Data analysis

First, the acupuncture group and control group were analyzed for comparability according to the baseline characteristics, including age, gender, smoking history, hypertension history, and SF-36. Student’s *t* test, Pearson’s Chi-square test or Fisher’s exact test were used to assess categorical variables. Descriptive analysis and statistical hypothesis testing were conducted with the SPSS software (SPSS 18.0).

## Result

Participant recruitment: All study participants, from emergency department (ED), were evaluated to exclude emergent condition and central nervous system disorder, such as infarction, hemorrhage, and aneurysm. Sixty participants (21–89 years old, 20 men and 40 women) were recruited into the study and divided into acupuncture group (*n* = 37) and control group (*n* = 23). In the present study, the statistical power (assuming α = 0.05) reached 96.4% for the variation of VAS between experimental and control groups after 30 min treatment.

**Fig. 2:**
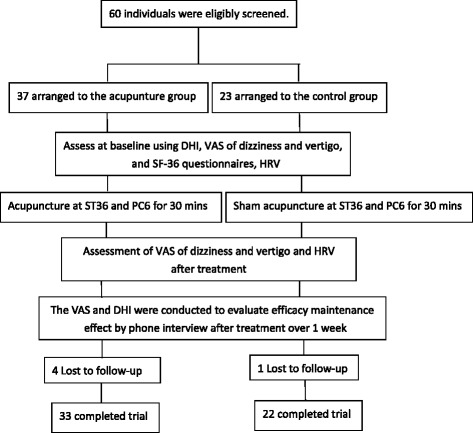
Experimental flowchart

The VAS and DHI were conducted to evaluate efficacy maintenance effect by phone interview after treatment over 1 week, *ref.* Fig. 2. There were 4 and 1 participants lost to follow-up in the acupuncture and control groups, respectively.

Baseline characteristics: Table 1 showed baseline participants characteristics, including age, gender, smoking history, hypertension history, body mass index, blood pressure and SF-36. There was no significant difference between two groups in baseline assessment.

**Table 1:** Baseline participant characteristics

Characteristics	Control group (*n* = 23)	Acupuncture (*n* = 37)	*p*-value
Age			
Age < 65y/o, No.(%)	10(43.5%)	24(64.9%)	0.10
Age≧65y/o, No.(%)	13(56.5%)	13(35.1%)	
Women, No.(%)	16(69.6)	24(64.9)	0.71
Smoking history, No.(%)	4(17.4)	8(21.6)	0.69
HTN history, No.(%)	10(43.5)	16(43.2)	0.99
BMI	23.58(4.41)	24.21(5.89)	0.69
systolic pressure, mean(SD)	121.3(32.16)	129.27(20.87)	0.25
diatolic pressure, mean(SD)	72.13(17.40)	75.7(7.7)	0.28
SF36			
PF, mean(SD)	70.65(31.38)	77.70(25.24)	0.34
RP, mean(SD)	46.74(49.03)	50.03(46.16)	0.68
BP, mean(SD)	54.74(26.29)	62.97(28.21)	0.31
GH, mean(SD)	41.52(21.05)	42.27(23.94)	0.89
VT, mean(SD)	53.26(19.16)	50.27(23.15)	0.61
SF, mean(SD)	73.37(22.39)	76.01(22.32)	0.66
RE, mean(SD)	63.77(48.11)	57.66(47.56)	0.63
MH, mean(SD)	54.43(16.30)	56.00(20.95)	0.76

*p*-value by independent *t* Test or Chi-quire test if needed. Phycial functioning (PF) Role limitation due to physical problems (RP), bodily pain (BP), general health (GH), and vitality (VT). Social functioning(SF) role limitation due to emotional problems(RE), and mental health (MH)

VAS of dizziness and vertigo and DHI assessment: Table 2 showed significant variation in pre-treatment and post-treatment VAS scores between control and acupuncture groups. The variation of VAS showed a significant difference (*p* = 0.001 and *p* = 0.037) between two groups after both 30 mins and 7 days. However, the variation of DHI showed no significant difference after 7 days.

**Table 2:** VAS of dizziness and vertig, DHI before and after acupuncture stimulation at PC6 and ST36

		Control group	Acupuncture	*p*-value
		(*n* = 23)	(*n* = 37)	
VAS				
	pre, mean(SD)	5.17 (2.08)	5.46 (1.91)	0.59
	Post (30mins), mean(SD)	4.83 (2.02)	4.49 (1.82)	0.50
	△VAS-1, mean(SD)	−0.35 (0.49)	−0.97 (0.80)	0.001*
	Post (7 days), mean(SD)	3.64 (2.46)	2.88 (1.85)	0.20
	△VAS-2, mean(SD)	−1.50 (1.63)	−2.48 (1.70)	0.037*
DHI				
	pre, mean(SD)	73.83 (14.21)	75.95 (11.11)	0.52
	post(7 days), mean(SD)	59.82 (17.93)	52.55 (19.11)	0.16
	△DHI, mean(SD)	−15.00 (14.18)	−23.45 (18.32)	0.073

P-value by independent *t* Test. △VAS-1 is the variation of VAS after 30mins; △VAS-2 is the variation of VAS after 7 days. △DHI is the variation of DHI after 7 days

*p < 0.05

Heart rate variability (HRV): Table 3 showed the parameters of HRV between pre-treat and post-treat in acupuncture group and control group. In acupuncture group, a significant difference (*p* = 0.03) was observed in HF, while HRV, HF%, LF%, LF/HF, VLF, LF RMSSD, PNN50 showed no significant change. The variation of HRV between two groups showed no significant difference, *ref.* Table 4.

**Table 3:** Parameters of Heart rate variability(HRV) after acupuncture stimulation at PC6 and ST36

	Control group		*p*-value	Acupuncture		*p*-value
	(*n* = 23)			(*n* = 37)		
	pre(*n* = 23)	post (*n* = 22)		pre(*n* = 37)	post (*n* = 33)	
HRV(SD)	52.4(46.4)	60.9(32.8)	0.62	74.4(68.2)	85.8(82.2)	0.39
HF%(SD)	52.6(16.4)	52.4(17.9)	0.66	47.5(18.5)	47.6(19.8)	0.90
LF%(SD)	47.2(16.4)	47.6(17.9)	0.66	52.5(18.5)	52.4(19.8)	0.90
LF/HF(SD)	1.1(0.8)	1.3(1.5)	0.92	1.5(1.3)	1.6(1.4)	0.81
VLF(SD)	3050.5(7699.5)	3355.1(4110.1)	0.61	4040.4(9305.8)	3612.3(4462.0)	0.74
LF(SD)	906.6(1972.5)	700.00	0.39	1596.7(2877.3)	1896.0(2579.2)	0.56
HF(SD)	544.2(731.0)	721.4(539.4)	0.87	830.8(769.1)	1082.7(1078.7)	0.03*
Total Power(SD)	2813.5(3778.9)	5055.7(5075.0)	0.81	6839.8(11156.8)	6713.(7423.0)	0.90
RMSSD(SD)	47.3(28.9)	62.8(25.7)	0.60	58.7(28.8)	59.4(33.3)	0.73
PNN50(SD)	19.8(22.8)	36.7(21.5)	0.35	32.0(23.8)	33.0(25.9)	0.69

*p*-value by paired *t* test comparing with post-treatment and pre-treatment. *p < 0.05

heart rate variability(HRV), high frequency (HF), low frequency (LF), very low frequency (VLF), sympatho-parasympathetic balance index (LF/HF), total power, root mean square of successive differences (RMSSD), and the proportion of NN50 divided by total number of NNs (PNN50)

**Table 4:** Changes in Heart rate variability (HRV) between control group and acupuncture group

	Control group	Acupuncture	*p*-value
	(*n* = 23)	(*n* = 37)	
△HRV(SD)	−13.50 (67.52)	11.06 (76.66)	0.33
△HF%(SD)	5.75 (32.52)	−0.29 (22.80)	0.49
△LF%(SD)	−5.75 (32.52)	0.29 (22.80)	0.49
△LF/HF(SD)	−0.04 (1.43)	0.05 (1.48)	0.81
△VLF(SD)	−2070.67 (11051.15)	−504.88 (8951.39)	0.63
△LF(SD)	−847.82 (2674.89)	166.03 (1568.84)	0.14
△HF(SD)	−78.36 (943.47)	229.35 (559.67)	0.20
△Total Power(SD)	−69.64 (6918.93)	−204.29 (9259.71)	0.97
△RMSSD(SD)	3.73 (30.93)	1.06 (17.28)	0.73
△PNN50(SD)	6.82 (24.94)	0.97 (14.05)	0.34

*p*-value by independent *t* test comparing with control group and acupuncture group

Safety and length of stay: No side effects were reported in this study. There was no bleeding, nausea, vomiting or other discomforts reported. The length of stay showed no significant difference (acupuncture group versus control group: 0.93 ± 0.87 days versus 0.87 ± 1.09 days).

## Discussion

Our study was designed to demonstrate that patients with dizziness and vertigo in ED can benefit from the application of acupuncture. Dizziness and vertigo were caused by an asymmetric involvement in the basal activity of the central and peripheral vestibular pathways. The causes of dizziness and vertigo are most likely to be benign positional vertigo, acute vestibular neuritis, and Ménière’s disease; however, vascular incidents and neurological causes must be kept in mind [20]. All participants were visited by otolaryngologist and neurologist to rule out vascular incidents and neurological causes to ensure participants’ safety in this study.

Acupuncture has been used for patients with Ménière’s disease and for relief of vertigo [21]. Neiguan (PC6) acupressure is useful in reducing dizziness and vertigo symptoms in patients affected by spontaneous and provoked vertigo [22]. One protocol was reported for chronic dizziness and assessment for 8 weeks, however, the trial was still undergoing and no concluding results reached yet [23]. Another study applied Baihui (GV20) and 4 spirit acupoints to improve balance function in stroke patients [8]. PC6 and ST36, the most frequently used acupoints in treating dizziness and vertigo, were regrettably not included in the test. The point PC6 (Nei-Guan) is one of the main points in pericardium meridian, and it is often stimulated for treating nausea, vomiting, and motion sickness. Another point ST36 (Zu-San-Li) is in stomach meridian, and it’s specific for the treatment of abdominal/epigastric pain, nausea, vomiting, hiccups, diarrhea, etc. Our study applied acupuncture on PC6 and ST36 and the results revealed immediate effect in reducing discomforts and VAS of both dizziness and vertigo significantly after 30 mins.

HRV measures the balance of autonomic nervous system which reflects physiological, hormonal, and emotional balance within our body [24]. Autonomic nervous system includes sympathetic and parasympathetic nervous system. Thus, HRV measures the balance of the activity of the sympathetic and parasympathetic nervous system. Changes in the HRV patterns provide a sensitive indicator of health impairments [25]. One study revealed autonomic nervous dysfunction relating to vertigo in Ménière’s disease as a predisposing factor [17]. Our study applied acupuncture to relieve dizziness and vertigo, and the corresponding changes in HRV were measured. The study showed HF increased significantly in acupuncture group. These results were inconsistent with the earlier report reviewed which showed significant decrease on HF and LF/HF ratio of HRV when acupuncture was performed on ST36 among healthy subjects and PC6 among both healthy and non-healthy subjects [24]. This discrepancy might be caused by differences in population selection, dizziness attack stage, and acupoints selection. More researches are required to determine if HRV can serve as an indicator for the therapeutic effect of acupuncture.

However, our study had several limitations. One limitation concerned the fact that patients from emergency department (ED) were recruited. Even though all patients were screened to exclude emergent conditions, yet no further classification regarding the types of dizziness and vertigo was performed. Another limitation concerned that the pilot study was not randomized and there are different number of participants between two groups. Participants were recruited in a standardized interview process. Participants could choose the experiment group or the control group to enroll study according to their willingness. Thus we found younger people would consider acupuncture as complementary therapy to relieve dizziness and vertigo rather than older people. Considering that acupuncture is a well-known means of medical treatment in the Chinese society, it is difficult to do blinded study about acupuncture. In order to minimize the bias from this, we used seed-patches on non-acupoints as sham acupuncture. The therapist did not performed any massage or press stimulation to prevent acupressure effect. Therefore, bias due to un-blinding approach cannot be ruled out.

A larger sample size would be indispensable to provide well-defined types of dizziness and vertigo for our evidence-based practice in future studies.

## Conclusion

Acupuncture revealed immediate effect of reducing discomforts and VAS of both dizziness and vertigo significantly after 30 min treatment. The results from this pilot study provided clinical evidence on the efficacy and safety of acupuncture to treat dizziness and vertigo in emergency department. In future work, a larger sample size study are required to provide evidence-based practice.
